# A novel quinoline derivative, DFIQ, sensitizes NSCLC cells to ferroptosis by promoting oxidative stress accompanied by autophagic dysfunction and mitochondrial damage

**DOI:** 10.1186/s12935-023-02984-w

**Published:** 2023-08-16

**Authors:** Yung-Ding Bow, Ching-Chung Ko, Wen-Tsan Chang, Sih-Yan Chou, Chun-Tzu Hung, Jau-Ling Huang, Chih-Hua Tseng, Yeh-Long Chen, Ruei-Nian Li, Chien-Chih Chiu

**Affiliations:** 1 PhD Program in Life Sciences, College of Life Science, Kaohsiung Medical University, Kaohsiung, 80708 Taiwan; 2 Department of Medical Imaging, Chi Mei Medical Center, Tainan, 71004 Taiwan; 3 Department of Health and Nutrition, Chia Nan University of Pharmacy and Science, Tainan, 71710 Taiwan; 4 Division of General and Digestive Surgery, Department of Surgery, Kaohsiung Medical University Hospital, Kaohsiung, 80708 Taiwan; 5 Department of Surgery, School of Medicine, College of Medicine, Kaohsiung Medical University, Kaohsiung, 80708 Taiwan; 6 Department of Biotechnology, Kaohsiung Medical University, Kaohsiung, 80708 Taiwan; 7 Department of Bioscience Technology, College of Health Science, Chang Jung Christian University, Tainan, 71101 Taiwan; 8 School of Pharmacy, College of Pharmacy, Drug Development and Value Creation Research Center, Kaohsiung Medical University, Kaohsiung, 80708 Taiwan; 9 Department of Medicinal and Applied Chemistry, Drug Development and Value Creation Research Center, Department of Medical Research, Kaohsiung Medical University Hospital, Kaohsiung Medical University, Kaohsiung, 80708 Taiwan; 10 Department of Biomedical Science and Environment Biology, Kaohsiung Medical University, Kaohsiung, 80708 Taiwan; 11 Department of Medical Research, Kaohsiung Medical University Hospital, Kaohsiung, 80708 Taiwan; 12 Department of Biological Sciences, National Sun Yat-Sen University, Kaohsiung, 80424 Taiwan; 13 Center for Cancer Research, Kaohsiung Medical University, Kaohsiung, 80708 Taiwan; 14 National Laboratory Animal Center, National Applied Research Laboratories, Taipei, 11571 Taiwan

**Keywords:** NSCLC, Ferroptosis, Chemotherapy, Camptothecin derivative, Mitochondrial dysfunction, Autophagic flux disruption, ROS imbalance

## Abstract

**Background:**

The development of nonapoptotic programmed cell death inducers as anticancer agents has emerged as a cancer therapy field. Ferroptosis, ferrous ion-driven programmed cell death that is induced by redox imbalance and dysfunctional reactive oxygen species (ROS) clearance, is triggered during sorafenib and PD-1/PD-L1 immunotherapy. DFIQ, a quinoline derivative, promotes apoptosis by disrupting autophagic flux and promoting ROS accumulation. Our pilot experiments suggest that DFIQ participates in ferroptosis sensitization. Thus, in this study, we aimed to reveal the mechanisms of DFIQ in ferroptosis sensitization and evaluate the clinical potential of DFIQ.

**Methods:**

We treated the non-small cell lung cancer (NSCLC) cell lines H1299, A549, and H460 with the ferroptosis inducer (FI) DFIQ and analyzed viability, protein expression, ROS generation, and fluorescence staining at different time points. Colocalization analysis was performed with ImageJ.

**Results:**

DFIQ sensitized cells to FIs such as erastin and RSL3, resulting in a decrease in IC_50_ of at least 0.5-fold. Measurement of ROS accumulation to explore the underlying mechanism indicated that DFIQ and FIs treatment promoted ROS accumulation and SOD1/SOD2 switching. Mitochondria, known ROS sources, produced high ROS levels during DFIQ/FI treatment. RSL3 treatment promoted mitochondrial damage and mitophagy, an autophagy-associated mitochondrial recycling system, and cotreatment with DFIQ induced accumulation of mitochondrial proteins, which indicated disruption of mitophagic flux. Thus, autophagic flux was measured in cells cotreated with DFIQ. DFIQ treatment was found to disrupt autophagic flux, leading to accumulation of damaged mitochondria and eventually inducing ferroptosis. Furthermore, the influence of DFIQ on the effects of clinical FIs, such as sorafenib, was evaluated, and DFIQ was discovered to sensitize NSCLC cells to sorafenib and promote ferroptosis.

**Conclusions:**

This study indicates that DFIQ not only promotes NSCLC apoptosis but also sensitizes cells to ferroptosis by disrupting autophagic flux, leading to accumulation of dysfunctional mitochondria and thus to ferroptosis. Ferroptosis is a novel therapeutic target in cancer therapy. DFIQ shows the potential to enhance the effects of FIs in NSCLC and act as a potential therapeutic adjuvant in ferroptosis-mediated therapy.

## Background

Non-small cell lung cancer (NSCLC) is a lung cancer subtype constituting 85% of lung cancer cases, including lung adenocarcinoma (LUAD), large-cell lung carcinoma (LCLC), and lung squamous cell carcinoma (LUSC) [1]. The therapeutic processes of NSCLC include chemotherapy, targeted therapy, and immunotherapy. Most of the therapeutic agents used for treatment are associated with programmed cell death, especially apoptosis, including cisplatin, vinorelbine, and paclitaxel [2–4]. However, chemoresistance is a critical issue during cancer therapy. Many factors are associated with chemoresistance, including the tumor microenvironment, ABC transporters, and apoptosis insensitivity [5–7]. Apoptosis insensitivity occurs when essential apoptotic genes are depleted by mutation, dysfunction, and silencing, which disrupts apoptotic flux and promotes cancer chemoresistance [8, 9]. Therefore, in recent years, nonapoptotic programmed cell death has become a popular therapeutic target in cancer therapy [10].

Nonapoptotic programmed cell death is an emerging field in anticancer therapy in which biological processes promote cell death in an orderly manner and are associated with several clinical cancer therapeutics [11, 12]. One of the nonapoptotic programmed cell death processes is ferrous ion-mediated cell death, ferroptosis, which contributes to PD-1/PD-L1 immunotherapy and the first-line hepatocellular carcinoma chemotherapy drug sorafenib [11, 12]. Ferroptosis is associated with ferrous ion accumulation, which results in consumption of the enzyme for reactive oxygen species (ROS) metabolism and promotes membrane lipid peroxidation [13]. In the last decade, ferroptosis has become a cancer therapeutic target, and several promising compounds have been developed for cancer therapy [14].

Quinoline derivatives, such as camptothecin and its derivatives, exhibit anticancer effects by acting as topoisomerase inhibitors that mediate DNA double-strand breaks (DSBs) during mitosis and disrupt the cancer proliferation process [15]. In previous studies, we developed the novel camptothecin derivative BPIQ and revealed its anticancer potential in several cancer types [16–18]. DFIQ is a novel synthetic quinoline compound that replaces the pyrrolidine domains of BPIQ with dimethylamine and fluorine. In a previous study, DFIQ showed anticancer effects by promoting apoptosis, ROS generation, and autophagic dysfunction [19]. Despite the high anticancer potential of DFIQ exhibited in NSCLC cells and zebrafish, the anticancer ability of DFIQ was tremendously deficient when administered at a lower dosage. As a result of the role of DFIQ in ROS imbalance, we examined the potential application of DFIQ in ferroptosis sensitization to promote ferroptosis at lower dosages and induce sensitivity to ferroptosis-targeted cancer therapy.

## Materials and methods

### Cell culture

Human NSCLC cell lines H1299, H460, and A549 were obtained from the American Type Culture Collection (ATCC; Manassas, VA, USA) and cultured in a 3:2 ratio of DMEM/F12 (Gibco; Thermo Fisher Scientific, Inc., Waltham, MA, USA) with 10% fetal bovine serum (FBS; SH30396, HyClone, Cytiva, Marlborough, MA, USA) and 1% P/S Solution (Penicillin‒Streptomycin; 30-002-CI, Corning, New York, USA). The cells were maintained at 37 °C with 5% CO_2_.

### Reagents

We utilized erastin (T1765, TargetMol, Wellesley Hills, MA, USA) and RSL3 (T3646 TargetMol, Wellesley Hills, MA, USA) as ferroptosis inducers (FIs). Liproxstatin-1 (HY-12726, MedChemExpress, NJ, USA) and acetylcysteine (NAC; A9166, Sigma‒Aldrich, Merck, Burlington, MA, USA) were utilized to offset ferroptosis and oxidative stress. The reagents, including DFIQ, erastin, RSL3 and liproxstatin-1, were dissolved in dimethyl sulfoxide (DMSO; D8418, Sigma‒Aldrich, Merck, Burlington, MA, USA) at concentrations of 10 mM, 20 mM, 10 mM and 10 mM, respectively.

### MTT assay

To measure cell viability, 6000 cells were seeded in 96-well plates and treated with DFIQ and FIs for 24 h. Cell viability was measured by the MTT assay. MTT (1.2 mM, Bio Basic, ON, Canada) was dissolved in culture medium and incubated at 37 °C with 5% CO_2_ for 4 h. 20% SDS with 0.01 M HCl was utilized to dissolve the formazan. The formazan absorbance was measured by a Synergy HTX Multimode Reader (BioTek, Santa Clara, CA, USA).

### Western blot analysis

The treated cells were lysed with RIPA lysis buffer (RB4475, Bio basic, ON, Canada) with proteinase and phosphatase inhibitors (K0010, K0021, MedChemExpress, NJ, USA), and the protein concentration was measured by a Dual-Range™ BCA Protein Assay Kit (Visual protein, TPE, Taiwan). We utilized SDS–polyacrylamide gel electrophoresis (SDS‒PAGE) to separate equal amounts of protein and then transferred the separated protein to polyvinylidene difluoride membranes (PVDF; IPVH00010, Millipore; Merck, DA, Germany). The transferred PVDF was then incubated with 5% nonfat milk in 1 × PBST (phosphate buffered saline with 0.1% Tween 20) for 1 h and hybridized with primary antibodies and HRP-conjugated secondary antibodies. HRP-mediated chemiluminescence was excited by the Immobilon ECL Ultra Western HRP Substrate (Millipore; Merck, DA, Germany). The blots were captured and analyzed by an Amersham imager 600 (GE Healthcare, Chicago, IL, USA).

### Antibody list

We utilized the following antibodies to perform Western blot analysis. The primary antibodies were anti-4-HNE (ARG23717, Arigo Biolaboratories, HSZ, Taiwan), anti-xCT (26864–1-AP, Proteintech, Rosemont, IL, USA), anti-GPX4 (ARG41400, Arigo Biolaboratories, HSZ, Taiwan), anti-GAPDH (ARG10112, Arigo Biolaboratories), anti-β-actin (NB600-501, Novus Biologicals, TPE, Taiwan), anti-catalase (219010, Merck, DA, Germany), anti-SOD2 (# 06–984, Merck), anti-SOD1 (ab51254, Abcam, EN, UK), anti-ULK1 (A8529, ABclonal, Woburn, MA, USA), anti-ND-1 (A5250, ABclonal), anti-UQCRC2 (A4181, ABclonal), anti-p62 (66184–1-Ig, Proteintech), and anti-Lamp2 (ab125068, Abcam). The secondary antibodies were anti-mouse IgG (7076, Cell Signaling, Danvers, MA, USA) and anti-rabbit IgG (7074, Cell Signaling).

### ROS detection

ROS accumulation was detected by dihydroethidium (DHE) (D11347, Thermo Fisher Scientific, Inc., Waltham, MA, USA) and 2’,7’–dichlorofluorescein diacetate (DCFDA) (10058, Biotium, Fremont, CA, USA) double staining. Briefly, the culture medium was replaced with medium containing 0.5 μM DHE, 10 μM DCFDA, and 15 μl/mL Hoechst 33342 (B2261, Sigma‒Aldrich, Merck, Burlington, MA, USA) for 30 min, and fluorescence was observed by microscopy as described in a previous study [19].

### Mitochondria and lysosome detection

The mitochondria and lysosomes were visualized by MitoTracker™ Green FM (M7514, Invitrogen, Thermo Fisher Scientific, Inc.), MitoTracker™ Red CMXRos (M7512, Invitrogen, Thermo Fisher Scientific, Inc.) and LysoTracker™ Red DND-99 (L7528, Invitrogen, Thermo Fisher Scientific, Inc.) and followed the manufacturer’s instructions. Briefly, the targeted cells were exposed to MitoTracker Green or MitoTracker Red, LysoTracker Red, and Hoechst 33,342 (B2261, Sigma‒Aldrich, Merck) for 30 min, washed and observed by microscopy or confocal microscopy as described in a previous study [19].

### Lipofuscin analysis

Lipofuscin was detected by Sudan black B staining (380B, Sigma‒Aldrich, Merck) following the manufacturer’s instructions. Briefly, the cells were fixed with 4% paraformaldehyde, stained with Sudan Black B solution, and finally labeled with Nuclear Fast red (1.15939, Sigma‒Aldrich, Merck). The cells were observed with a phase-contrast microscope.

### Statistical analysis

The experiments were performed at least in triplicate, and the differences between groups were analyzed by one-way analysis of variance (ANOVA) or t test. *p* < 0.05 was considered significant.

## Results

### DFIQ sensitized cells to FIs

**Fig. 1:**
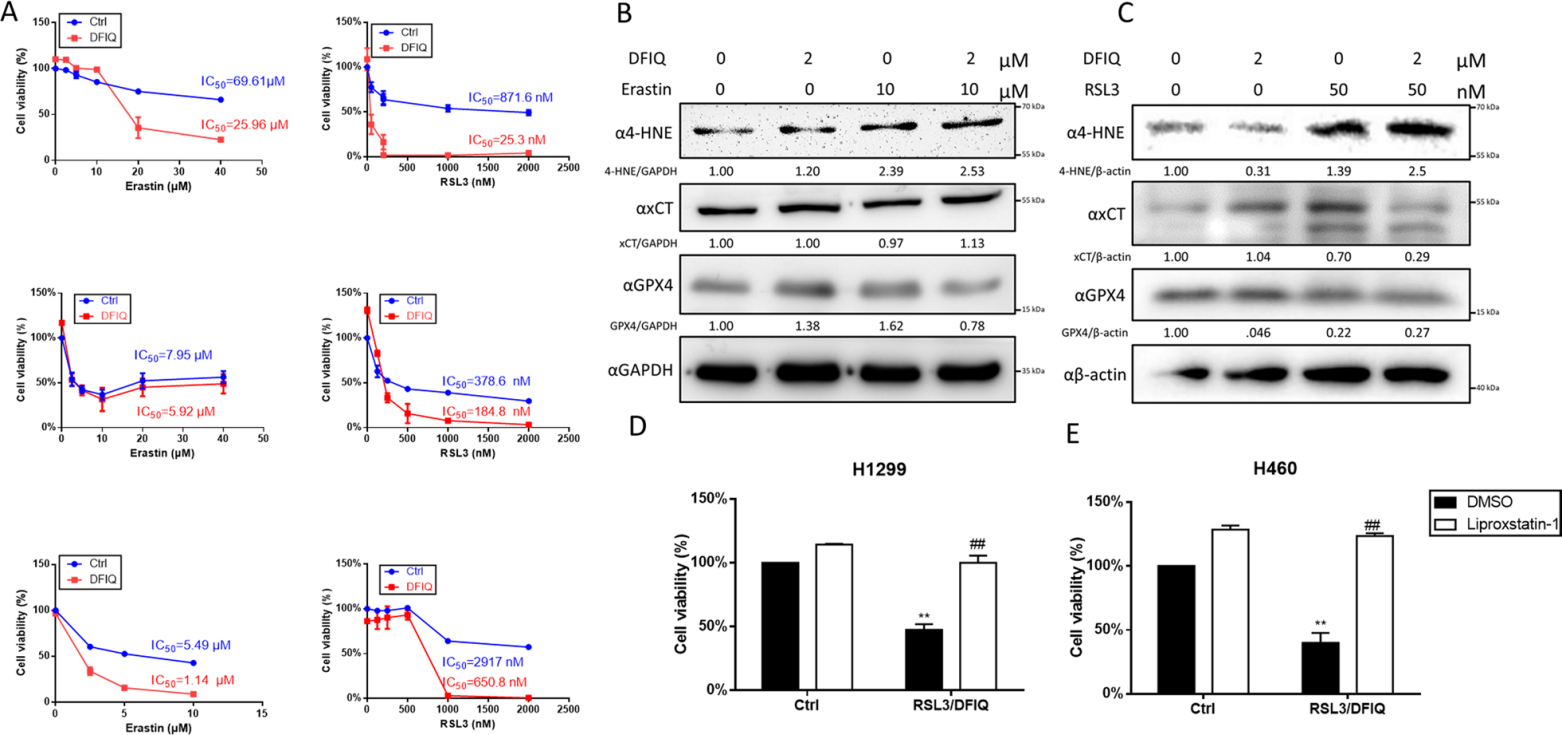
DFIQ sensitized NSCLC cells to FIs and promoted ferroptosis. **a** IC_50_ alterations of FIs after DFIQ treatment. n = 4. **b** and **c** Western blot analysis of ferroptosis-associated protein expression after DFIQ and FI (erastin **b** and RSL3 **c**) treatment in H1299 cell lines. **d** Changes in the viability of H1299 and H460 cells cotreated with DFIQ/RSL3 and the ferroptosis inhibitor liproxstatin-1. **p < 0.01 compared to the control groups, ##p < 0.01 compared with RSL3 only groups

In a previous study, we revealed the anticancer potential of DFIQ in NSCLC cell lines and the potential mechanisms by which ROS imbalance and autophagic dysfunction occur. Ferroptosis is an ROS-associated programmed cell death that involves ferrous ion-mediated ROS imbalance. We cotreated NSCLC cells with DFIQ at a concentration lower than the IC_50_ of DFIQ and the FIs erastin and RSL3. The results indicated that DFIQ promoted cell sensitivity to ferroptosis, decreased the IC_50_ values of erastin and RSL3, and promoted similar cell morphology (Fig. 1A). DFIQ treatment significantly decreased the IC_50_ values of the FIs erastin and RSL3 in H1299 and A549 cells and sensitized H460 cells to RSL3 (Fig. 1B and Table 1). To investigate the role of ferroptosis in cell death mediated by DFIQ and FIs, ferroptosis protein markers such as GPX4 depletion and 4-HNE accumulation were discovered after DFIQ/FI cotreatment. However, the expression of xCT did not show consistent changes after DFIQ/FI treatment (Fig. 1C and D). Liproxstatin-1 is an inhibitor of ferroptosis, arresting the accumulation of lipid hydroperoxides [20]. The cells treated with RSL3 and DFIQ were subjected to liproxstatin-1 administration, and cell death caused by RSL3/DFIQ cotreatment was inhibited (Fig. 1E). The results indicated that DFIQ treatment sensitized cells to FIs, and relatively low concentrations of FIs were necessary to activate ferroptosis in NSCLC cell lines.

**Table 1:** IC_50_ of FIs after DFIQ treatment

Cell	FIs	IC_50_				FC IC_50_
		Ctrl	95%CI	DFIQ	95%CI	
H1299	Erastin	69.61 μM	69.61 ~ 79.66 μM	25.96 μM	11.51 ~ 58.57 μM	0.37
	RSL3	817.7 nM	556.4 ~ 1365 nM	25.31 nM	18.2 ~ 35.2 nM	0.03
H460	Erastin	7.60 μM	5.327 ~ 11.89 μM	5.93 μM	4.086 ~ 8.596 μM	0.78
	RSL3	378.6 nM	294.1 ~ 487.4 nM	184.8 nM	128.5 ~ 265.6 nM	0.49
A549	Erastin	5.48 μM	4.00 ~ 7.532 μM	1.14 μM	0.92 ~ 1.41 μM	0.21
	RSL3	2917.0 nM	1869 ~ 4551 nM	650.8 nM	238.9 ~ 1772 nM	0.22

*FIs* ferroptosis inducers, *IC*_*50*_ half maximal inhibitory concentration, *95% CI* 95% confidence intervals, *FC* fold change. N = 4

### ROS played a potential role in DFIQ-induced ferroptosis

**Fig. 2:**
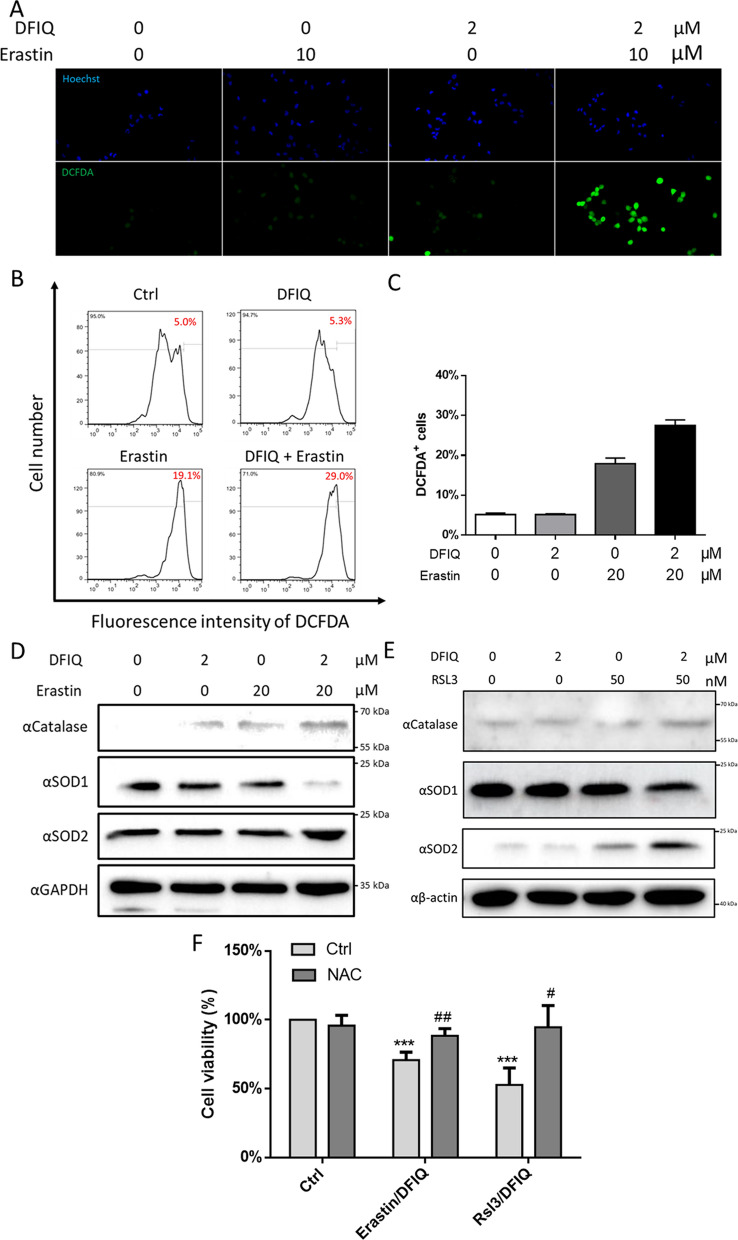
DFIQ/FIs mediated ROS accumulation and promoted ferroptosis. **a** DCFDA/DHE staining of H1299 cells after erastin/DFIQ treatment. **b** Flow cytometry analysis of H1299 cells after erastin/DFIQ treatment. **c** Quantification of **b**. **d** and **e** Western blot analysis of ROS catalytic protein expression after DFIQ and erastin **b** treatment or RSL3 **c** treatment. **d** H1299 viability changes after pretreatment with NAC and DFIQ/FIs. ***p > 0.001 compared to control, ##*p* > 0.01 #*p* > 0.05 compared with DFIQ/FIs only groups

ROS are involved in many biological processes and play a vital role in ferroptosis [21]. ROS metabolism imbalance results in ROS accumulation and promotes ferroptosis by inducing lipid peroxidation. This process escalates the load of the ROS removal system, which includes GPX4, an essential protein that removes the oxidative stress caused by ferrous ions [21]. Thus, oxidative stress was observed through DHE and DCFDA double staining to measure the enrichment of O_2_^−^ and H_2_O_2_ after erastin and DFIQ treatment. Treatment with erastin or DFIQ promoted O_2_^−^ and H_2_O_2_, respectively (Fig. 2A, B and C), and induced ROS metabolism protein expression (Fig. 2D and E). To further clarify the mechanism of ROS in DFIQ-sensitized ferroptosis, the antioxidant acetylcysteine (NAC) was used to remove ROS, and differences after DFIQ/FI treatment were observed [22]. Pretreatment with NAC restored the cell death initiated by erastin/RSL3 and DFIQ treatment (Fig. 2D). The results indicated that cotreatment with FIs and DFIQ elevated intracellular ROS accumulation and promoted oxidative stress in NSCLC cells.

### DFIQ exerted the potential to promote mitochondrial damage and accumulation

**Fig. 3:**
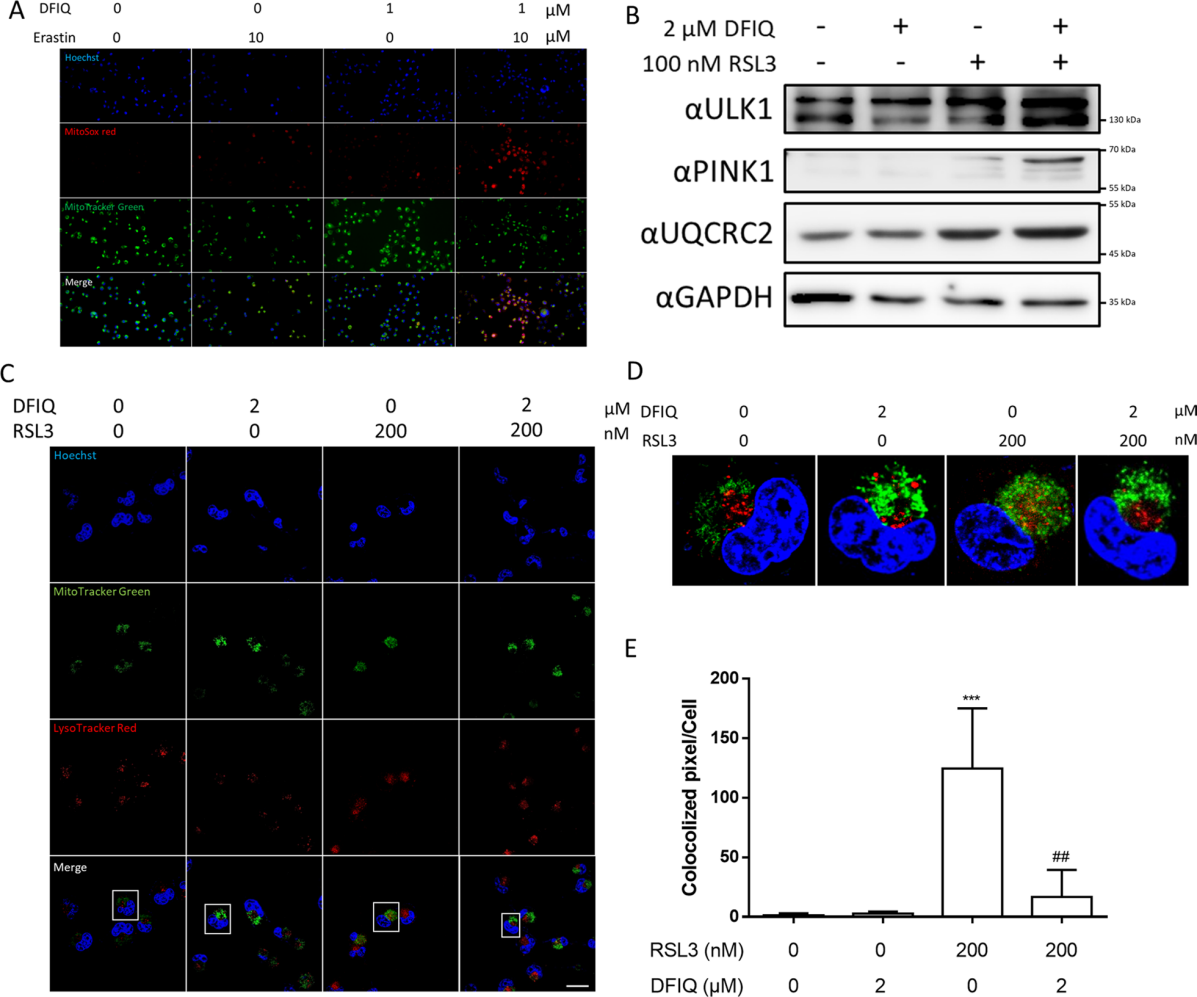
DFIQ/FI treatment mediated mitochondrial damage and mitophagy dysfunction. **a** MitoSOX Red/MitoTracker Green staining after DFIQ and erastin treatment. **b** Western blot analysis of mitochondrial damage proteins and mitochondrial protein expression after 6 h of DFIQ and RSL3 treatment. **c** Confocal microscopy analysis of H1299 cells stained with LysoTracker Red/MitoTracker Green after 6 h of DFIQ and RSL3 treatment. The scale bar is indicated as 20 μm. **d** Enlarged image of **c**. **e** The colocalization of lysosomes (red) and mitochondria (green) in **c** was quantified by ImageJ. ***p < 0.001 compared with the control, ##*p* < 0.01 compared with the 200 nM RSL3 group

Mitochondria are one of the major sources of ROS [23]. Mitochondrial dysfunction, especially in the electron transport chain, results in tremendous oxidative stress [24]. Thus, mitochondrial ROS might play a role in the ROS accumulation caused by erastin/DFIQ cotreatment. MitoSOX Red was employed to observe the production of ROS in mitochondria. Compared to the individual treatments with erastin and DFIQ, as well as the control, the cotreatment induced significant increases in mitochondrial ROS levels in NSCLC cells (Fig. 3A).

PINK1 is a Ser/Thr kinase associated with the detection of mitochondrial damage and is constitutively degraded by mitochondrial proteases in healthy mitochondria. When mitochondria are damaged, resulting in the loss of mitochondrial membrane potential (ΔΨ), PINK1 stabilizes and accumulates at the outer mitochondrial membrane, phosphorylating downstream parkin proteins to activate mitophagy [25]. The accumulation of PINK1 and subsequent clearance play vital roles in mitochondrial quality control. On the other hand, ULK1 is a critical kinase involved in the activation of mitophagy [26]. ULK1 receives the stress signal from ROS-activated AMPK and promotes the generation of the phagophore, initiating mitophagy to remove damaged mitochondria [27, 28]. Both PINK1 and ULK1 were upregulated after RSL3/DFIQ treatment, indicating an increase in mitochondrial damage and the activation of mitophagy (Fig. 3B). The total accumulation of mitochondria was also measured by observing OXPHOS complex expression. Interestingly, the UQCRC2 protein, known as a part of mitochondrial complex III, was upregulated, which indicated an increase in mitochondria after DFIQ/RSL3 treatment (Fig. 3B).

The activation of mitophagy recycles damaged mitochondria and decreases the number of mitochondrial proteins [29]. The results indicated that DFIQ/RSL3 treatment initiated mitophagy but promoted the accumulation of mitochondria. Thus, mitophagy dysfunction is a potential event that induces the accumulation of mitochondria. To evaluate the process of mitophagy, the lysosomes and mitochondria were labeled and observed by confocal microscopy (Fig. 3C). The results showed that lysosomes and mitochondria were fused during RSL3 treatment and separated when cotreated with DFIQ (Fig. 3C–E). In our previous study, high doses of DFIQ administration inhibited autophagosome fusion and lysosome fusion. Furthermore, mitophagy also utilizes autophagy to finalize mitochondrial recycling. Overall, the data indicated that treatment with FIs induced mitochondrial damage and mitigated mitophagy to eliminate damaged mitochondria. DFIQ treatment disrupted mitophagy by interfering with the fusion of damaged mitochondria and lysosomes.

### DFIQ mediated autophagic dysfunction during FI treatment

**Fig. 4:**
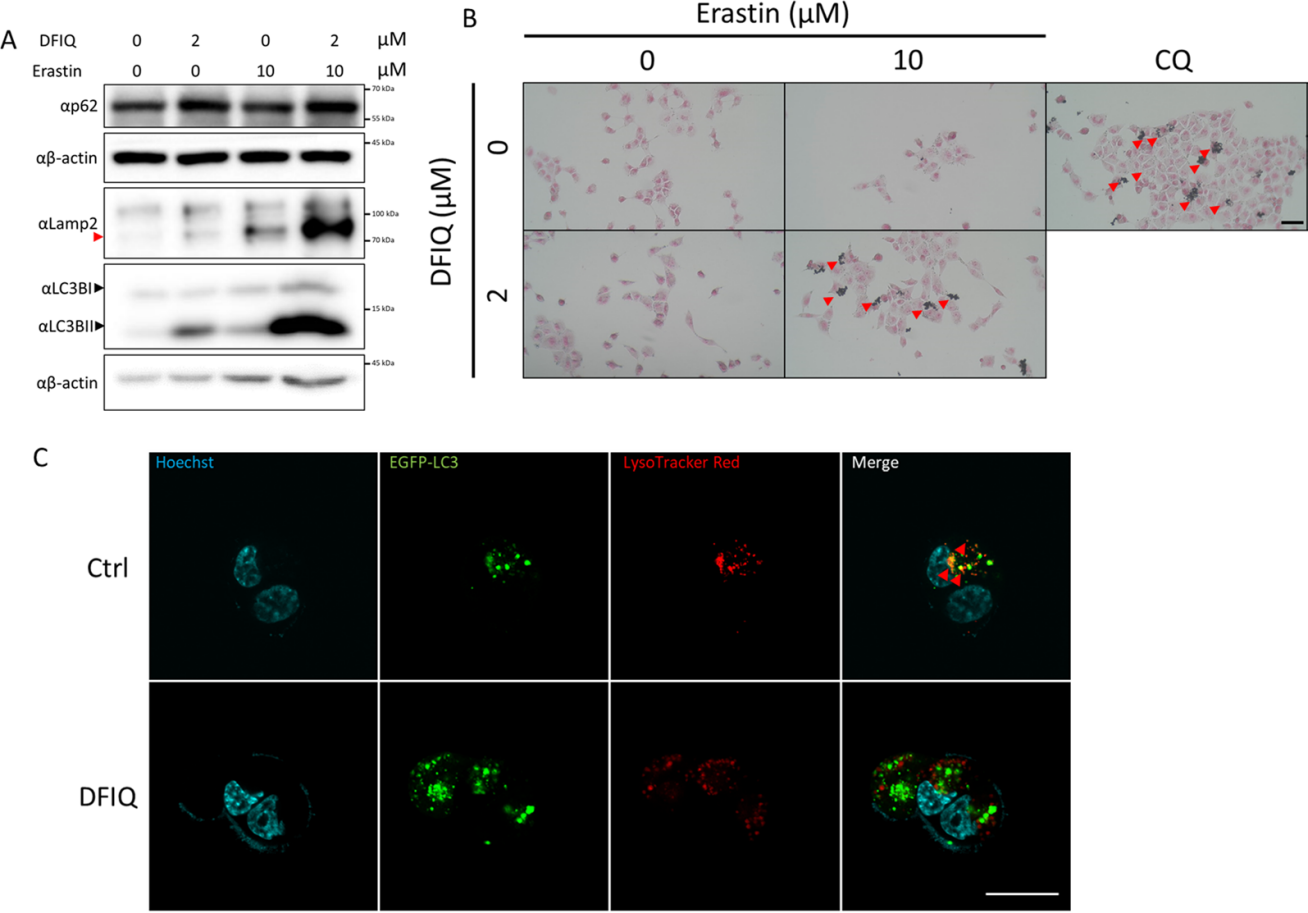
DFIQ treatment promoted autophagic flux disruption. **a** Western blot analysis of autophagic protein expression after DFIQ/erastin treatment. **b** Lipofuscin accumulation assay during DFIQ/erastin treatment. Red arrows indicate the accumulation of lipofuscin. CQ: chloroquine, a positive control to inhibit autophagosome and lysosome fusion. The scale bar indicates 100 μm. **c** Confocal microscopy analysis of 2 μM DFIQ-treated H1299 cells overexpressing EFGP-LC3B and stained with LysoTracker Red. Red arrows indicate autophagolysosomes. The scale bar indicates 20 μm

Autophagy is a stress response to clean damaged organelles, proteins, and intracellular fragments [30]. Autophagy also plays a role in ROS clearance by engulfing and degrading ROS [31, 32]. The autophagic protein LC3B was significantly lipidated and transformed into LC3BII when erastin and DFIQ were used together, indicating the initiation of autophagy (Fig. 4A). However, interestingly, p62 and Lamp2, which are considered to be consumed during autophagic flux, were upregulated during erastin/DFIQ cotreatment (Fig. 4A). The alteration suggested that autophagic flux was disrupted, inducing autophagic protein accumulation. We further investigated autophagic flux disruption with a lipofuscin accumulation assay. The results indicated that erastin and DFIQ treatment promoted lipofuscin accumulation and indicated the potential function of DFIQ in disrupting autophagic flux (Fig. 4B). To further investigate the fusion of autophagosomes and lysosomes, we overexpressed LC3B with EGFP, located lysosomes with LysoTracker Red staining, and observed colocalization with confocal microscopy after erastin and DFIQ treatment. Figure 4C indicates that DFIQ treatment disrupted autophagolysosome generation by inhibiting autophagosome and lysosome fusion.

### DFIQ sensitized cells to the clinical anticancer agent

**Fig. 5:**
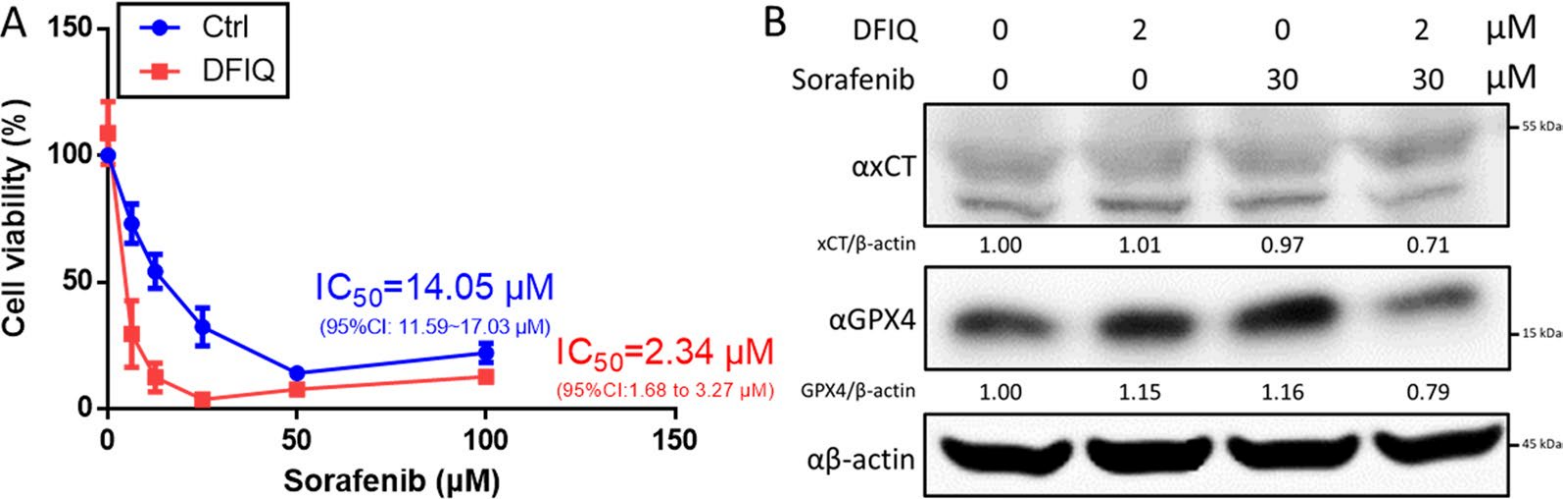
DFIQ sensitized NSCLC cells to the clinical drug sorafenib and promoted ferroptosis. **a** Cell viability alteration of sorafenib-treated H1299 cells after DFIQ administration for 24 h. **b** Western blot analysis of ferroptotic protein expression after treatment of H1299 cells with sorafenib and DFIQ

Ferroptosis was found to play a role in several clinical therapies, including sorafenib, a first-line treatment for HCC, and PD-1/PD-L1 immunotherapy [11, 12, 33]. To evaluate the interaction of DFIQ with other clinical therapeutics, we observed the effect of DFIQ during sorafenib treatment. The results indicated that DFIQ treatment decreased the sorafenib IC_50_ from 14.05 μM to 2.34 μM (Fig. 5A). In addition, the ferroptotic proteins xCT and GPX4 were exhausted after sorafenib and DFIQ treatment (Fig. 5B). The results provide a potential application of DFIQ in clinical usage that utilizes DFIQ as an adjuvant reagent to improve the efficiency of clinical ferroptotic therapy.

## Discussion

Ferroptosis is a novel therapeutic target for cancer and was discovered through sorafenib therapy and PD-1/PD-L1 immunotherapy [11, 12]. Sorafenib was shown to promote ferroptosis in several cell types by inhibiting xCT activation, a cystine/glutamate antiporter that provides cysteine to the GSSG/GSH antioxidant cycle, which plays a vital role in ferrous ion metabolism [33]. Ferroptosis is associated with many biological and therapeutic processes. This study investigated the role of a novel synthetic small molecule, DFIQ, in sensitizing cells to ferroptosis. DFIQ treatment induced ROS generation, disrupted autophagic flux, and promoted cell apoptosis at relatively high concentrations [19]. In addition, DFIQ sensitized cells to ferroptosis, tremendously decreasing the IC_50_ of FIs, including erastin, RSL3, and sorafenib. Upon comparing MRC-5 lung fibroblasts to NSCLC cells, we found no evidence of DFIQ's selective cytotoxicity against the former (data not shown). Fortunately, with the advantages of nanoparticle development, DFIQ can be further encapsulated into nanocarriers conjugated with ligands that can bind to cancer cells overexpressing membrane receptors or other tumor antigens [1], whichmay increase the specificity and cytotoxicity of DFIQ against lung cancer. For example, poly(ethylene glycol)-poly(lactic-co-glycolic acid) (PEG-PLGA) conjugated with folic acid (FA) as a nanocarrier has been reported to selectively deliver the encapsulated anticancer drugs cisplatin and paclitaxel and cause cytotoxicity in NSCLC M109 (FA receptor-positive) cells [34]. The data indicate that DFIQ regulates ferroptosis sensitivity in NSCLC cells and suggest the potential of DFIQ as a potent clinical therapy for cancer.

Oxidative stress is a critical factor during ferroptosis initiation [35]. Treatment with FIs or DFIQ promoted ROS metabolism imbalance and immense ROS accumulation. The accumulation of ROS is associated with most programmed cell death processes, including apoptosis, necroptosis, and ferroptosis. [36–38]. DFIQ with FIs elevated oxidative stress at a relatively low concentration compared to the IC_50_ of DFIQ alone, as shown in Fig. 2, and SOD family modifications may be a mechanism by which DFIQ sensitizes cells to FIs. Three major SOD isoforms were discovered in mammals: SOD1, a cytosolic dismutase conjugated with copper and zinc; SOD2, a mitochondrial dismutase conjugated with manganese; and SOD3, an extracellular SOD conjugated with copper and zinc [39]. SOD modifications have been discovered in many biological processes and anticancer treatments and are associated with ROS imbalance [40–43]. Luena Papa et al. found that SOD2 levels were decreased and SOD1 levels were increased during breast cancer progression [41]. In our previous study, SOD1 transformed into SOD2 during C_8_-ceramide treatment, and ROS imbalance and apoptosis were measured in H1299 cells [42]. Generally, SOD1 is a cytosolic SOD and balances cytosolic ROS, while SOD2 catalyzes mitochondrial ROS. SOD transformation indicated that SOD1 levels decreased and SOD2 levels increased during therapeutic DFIQ/FIs and C_8_-ceramide treatment [42]. Moreover, mitochondria are the primary generators of ROS, and ROS leakage mediates several injury response mechanisms, including many carcinogenesis pathways, which indicates that SOD activity is a potential therapeutic target during cancer development and therapy [44–46].

Mitochondrial quality control is a vital intracellular mechanism that utilizes autophagy to remove damaged mitochondria and is called “mitophagy” [47]. Damaged mitochondria exhibit many features, such as mitochondrial DNA damage, membrane permeabilization, ROS accumulation, and OXPHOS dysfunction. Mitochondrial damage can be detected by measuring the expression of PINK1 and ULK1. PINK1, a Ser/Thr kinase, is unstable in healthy mitochondria due to PINK1 C-terminal transport into the inner mitochondrial membrane (IMM) and is degraded by the mitochondrial proteinases MPP and PARL. Damaged mitochondria lose their membrane potential, and the transport of PINK1 is blocked, causing the accumulation of PINK1 and promoting downstream E3 ubiquitin ligase, mediating parkin protein phosphorylation and activation, recruiting autophagic receptors and triggering mitophagy [48, 49]. J Zhang et al. discovered that the activation of mitophagy mediated the degradation of mitochondrial proteins via elevated lysosomal function [29]. Figure 3B indicates the upregulation of PINK1 and ULK1, which caused mitochondrial damage and activation of mitophagy after RSL3 treatment. In addition, the mitochondrial protein UQCRC2 accumulated. Mitophagy clearance mediated the downregulation of mitochondrial proteins, and the results indicated that mitophagic flux dysfunction caused damaged mitochondrial accumulation and promoted ROS accumulation.

## Conclusions

**Fig. 6:**
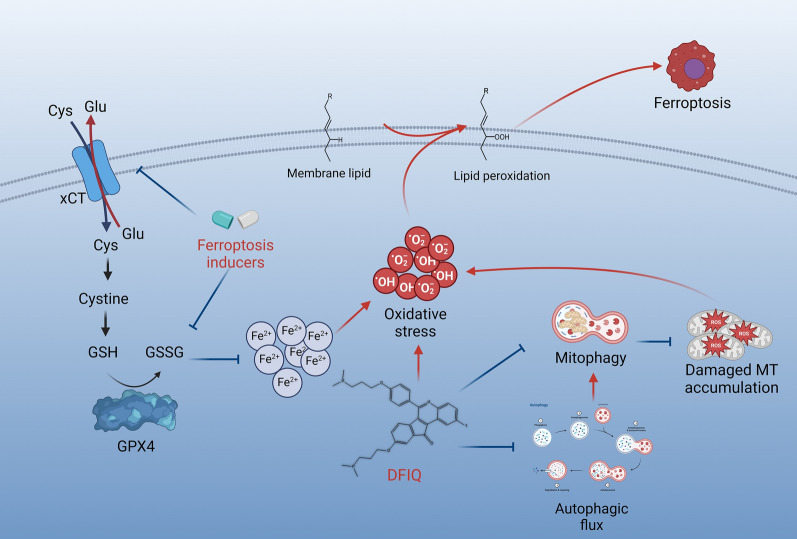
Illustration of the study results. Ferroptosis plays a vital role in oxidative stress-mediated cell death. When oxidative stress is triggered by FIs, autophagy and mitophagy are activated, ROS are removed, and damaged mitochondria are cleared. After DFIQ treatment, autophagic flux was disrupted. Moreover, mitophagy was also disrupted, promoting damaged mitochondria accumulation, elevating ROS and oxidative stress, and ferroptosis

Ferroptosis is a novel target in cancer therapy. In this study, we discovered a new application of the synthetic quinoline small molecule DFIQ, which acted as a ferroptosis sensitizer that promoted cell death with low concentrations of FIs, including erastin, RSL3, and sorafenib. ROS accumulation due to mitochondrial and autophagic dysfunction mediated by DFIQ played a potential role in the ferroptosis process (Fig. 6). In conclusion, DFIQ has the potential to serve as an adjuvant therapeutic reagent in ferroptosis-mediated therapy.

## Data Availability

The datasets supporting the conclusions of this article are included within the article.

## Abbreviations

DCFDA: 2’,7’–Dichlorofluorescein diacetate
DFIQ: 9-[3(Dimethylamino)propoxy]-6-{4-[3-(dimethylamino)propoxy]phenyl}-2-fluoro-11H-indeno[1,2-c]quinolin-11-one
DHE: Dihydroethidium
DSBs: DNA double-strand breaks
FI: Ferroptosis inducer
GPX4: Glutathione peroxidase 4
IC_50_: Half-maximal inhibitory concentration
Lamp2: Lysosome-associated membrane protein-2
LC3B: Tubule-associated protein 1 light chain 3 beta
LCLC: Large-cell lung carcinoma
LUAD: Lung adenocarcinoma
LUSC: Lung squamous cell carcinoma
NSCLC: Non-small cell lung cancer
OXPHOS: Oxidative phosphorylation
PD-1: Programmed death-1
PD-L1: Programmed cell death-ligand 1
PINK1: PTEN-induced kinase 1
ROS: Reactive oxygen species
SOD: Superoxidase dismutase
ULK1: Unc-51 like autophagy activating kinase 1
UQCRC2: Ubiquinol-cytochrome C reductase core protein 2
